# Transcriptome-Wide Analysis and Functional Verification of RING-Type Ubiquitin Ligase Involved in Tea Plant Stress Resistance

**DOI:** 10.3389/fpls.2021.733287

**Published:** 2021-10-21

**Authors:** Dawei Xing, Tongtong Li, Guoliang Ma, Haixiang Ruan, Liping Gao, Tao Xia

**Affiliations:** ^1^State Key Laboratory of Tea Plant Biology and Utilization, Anhui Agricultural University, Hefei, China; ^2^School of Life Sciences, Anhui Agricultural University, Hefei, China

**Keywords:** *Camellia sinensis*, RING-containing proteins, E3 ubiquitin ligase, MIEL1, biological and abiotic stress

## Abstract

The ubiquitin/26S proteasome pathway is a critical protein-degradation pathway in plant growth and development as well as in nearly all biological and abiotic stress processes. Although as a member of the ubiquitin/26S proteasome pathway, the E3 ubiquitin ligase family has been shown to be essential for the selective degradation of downstream target proteins, it has been rarely reported in tea plants (*Camellia sinensis*). In this study, through database searches and extensive manual deduplication, 335 RING finger family proteins were selected from the Tea Plant Information Archive. These proteins were divided into six categories by the difference of RING finger domain: RING-H2, RING-HCa, RING-HCb, RING-C2, RING-v, and RING-G. Stress-induced differential gene expression analysis showed that 53 proteins in RING finger family can respond to selected exogenous stress. *In vitro* ubiquitination assays indicated that TEA031033, which was named CsMIEL1, exhibited the activity of E3 ubiquitin ligases. *CsMIEL1-overexpressing* transgenic *Arabidopsis thaliana* seedlings were resistant to some exogenous abiotic stresses, such as salt and drought stress but sensitive to exogenous methyl jasmonate treatment. Furthermore, CsMIEL1 reduced the accumulation of anthocyanin in transgenic plants in response to low temperature treatment. The results of this article provide basic date for studying the role of ubiquitin/26S proteasome pathway in tea plants response to stresses.

## Introduction

Tea plant [*Camellia sinensis* (L.) O. Kuntze], a perennial evergreen woody plant, encounters various abiotic (drought, heat, cold, and salt) and biological stresses (pests, viruses, and herbivore foraging). Under these stresses, plants undergo various degrees of damage. For example, under high salt stress, an imbalance of Na^+^/K^+^ inside and outside the cell restricts plant growth and accumulates excessive reactive oxygen species, which causes osmotic and even oxidative stresses (Munns and Tester, 2008). Through long-term natural selection, plants have evolved various physiological and biochemical defense mechanisms to deal with stresses (Hou et al., 2009), such as antioxidant defense mechanisms (Dai and Mumper, 2010), apoptosis (Jones, 2001), and autophagy (Avin-Wittenberg, 2019). Plants can tolerate a harsh environment and maintain normal growth, in recent years, more and more researches have begun to pay attention to how the ubiquitination process of plants works under various stresses.

The 26s-proteasome-mediated ubiquitination degradation pathway (ubiquitin/26s proteasome) is central to apoptosis (Kurepa et al., 2012). The ubiquitination of substrate proteins requires the participation of E1-ubiquitin-activating, E2-ubiquitin-conjugating enzymes, and E3-ubiquitin ligases. First, the cysteine residue (Cys) on the active site of E1 is covalently bound to the terminal glycine of the ubiquitin molecule through a high-energy thioester bond, and Adenosine 5′-triphosphate (ATP) is consumed to activate the ubiquitin (Stone, 2014). Second, the ubiquitin is transferred to the Cys residue of E2 to form an E2-Ub thioester complex. Finally, E3 transfers the ubiquitin from E2 to the substrate protein (Downes et al., 2003). After that, the 26S proteasome degrades the ubiquitinated-modified substrate protein, and the deubiquitinating enzyme hydrolyzes and releases the ubiquitin, which triggers the ubiquitination modification process.

As a key of the ubiquitin-proteasome pathway, E3 ligases are critical in the recognition of target substrates. Because of the importance of E3 ligase, the genes encoding E3 are the most numerous in the same plant genome. For example, in the *Arabidopsis thaliana* genome, approximately 6% of the genes are related to the ubiquitin/26s proteasome pathway (Fu et al., 1999; Yan et al., 2000; Bachmair et al., 2001; Gagne et al., 2002; Kosarev et al., 2002). Two genes encode E1 and are the least numerous. At least 37 genes encode E2 and E2-like proteins, but more than 1,400 genes encode E3 (Smalle and Vierstra, 2004; Mazzucotelli et al., 2006; Vierstra, 2009). E3 ubiquitin ligases can be divided into HECT, Ubox, and RING types, but RING-type ligases can be additionally divided into complex and simple types (Vierstra, 2003). The Cys-rich RING protein was originally named the “*Really Interesting New Gene*” based on its unique domain (Freemont, 1993), the structure of the RING finger domain that binds a pair of zinc atoms is conserved (Yang et al., 2019), and eight conserved docking sites in the domain exist for binding the ubiquitin-E2 intermediate during ubiquitin transfer (Barlow et al., 1994; Borden et al., 1995; Zheng et al., 2000). The RING finger domain is similar to the well-known Zinc-finger domain in structure; however, the Zinc-finger domain functions in the form of DNA-protein binding, whereas the RING-finger domain functions in the form of a protein–protein interaction (Nardelli et al., 1991).

In previous studies, it has been shown that more than 70% of RING-containing proteins in *Arabidopsis thaliana* act as an E3 ubiquitin ligase (Stone et al., 2005). For example, AtATL78 is a RING-type E3 ubiquitin ligase located in the plasma membrane, which promotes ABA-dependent plant stomata closure, and also can participate in cold and drought stress responses. In a study of cold stress response, it was found that compared with the wild-type plants, the transcription levels of cold-stress-induced genes *RD29A*, *RD29B*, *RD20*, and *P5CS1* were significantly upregulated in *atl78* mutants, and lower H_2_O_2_ accumulation was detected, which indicated that AtATL78 was a negative factor in cold stress regulation (Suh et al., 2016). Research has also investigated the RING-H2 type protein MYB30-INTERACTING E3 LIGASE 1 (MIEL1). In *Arabidopsis*, MIEL1 mediates the degradation of the transcription factor MYB30, which acts as a positive regulator of the hypersensitive cell death program in plants, and it can interact with AtMIEL1 (Marino et al., 2019), the interaction between MIEL1 and MYB30 affected the formation of the plant’s waxy layer, weakens the plant’s defense ability (Lee et al., 2017), and MIEL1 also negatively regulates ABA signaling by promoting MYB96 turnover (Lee and Seo, 2016). In apples, MdMYB308L is a known target gene identified to interact with MIEL1, it positively regulates cold tolerance and anthocyanin accumulation by interacting with MdbHLH33 and enhancing its binding to MdCBF2 and MdDFR promoters. When MIEL1 interacts with MYB308L, MdMIEL1 directly degrades MdMYB308L through the 26S-proteasome-mediated ubiquitination degradation pathway, thereby reducing the cold tolerance and anthocyanin accumulation promoted by MdMYB308L (An et al., 2017).

Although numerous studies have concluded that RING domain–containing proteins have multiple regulatory roles in plant growth and development, few studies have investigated the function of RING finger family proteins in tea plants. In this study, RING-containing proteins in tea plants were selected from the Tea Plant Information Archive (TPIA), and the predicted responses of RING domain proteins to abiotic and biotic stresses were explored. One of them was proven to have a ubiquitination function and likely contributing to the resistance of *A. thaliana* to abiotic stress.

## Materials and Methods

### Identification of RING Domain-Containing Proteins in the Tea Plant

We downloaded the sequence of 477 RING-containing proteins that have been reported to be present in *Arabidopsis* (Stone et al., 2005) from The Arabidopsis Information Resource (TAIR)^1^ and constructed their domains in Pfam^2^. Six different Hidden Markov model (HMM) motifs were collected. HMM-Blast was applied to the protein sequences in the database downloaded from TPIA^3^ with HMMer^4^ (Mistry et al., 2013). For the prediction and analysis of the domains of these proteins, we used InterPro^5^ for the initial appraisal. Pfam (see text footnote 2) and SMART^6^ for a secondary verification and determination of redundant domains.

### Expression Analysis of RING Finger Family Genes in the Tea Plant

We downloaded the expression levels of these RING family genes under different stresses from TPIA (Supplementary Table 1). The gradient of cold treatment was CA1-6h (10°C for 6 h), CA1-7d (4–10°C for 7 days), CA2-7d (0–4°C for 7 days), and DA-7d [recovery afterward at room temperature (20–25°C) for 7 days] (Wang et al., 2013). Salt and drought treatments were conducted for 24, 48, and 72 h (Zhang et al., 2017). MeJA treatment was applied in gradients of 12, 24, and 48 h (Shi et al., 2015). We calculated the relative ratio of these expression levels to the control, which was normalized by log2 (Supplementary Figure 1). When the expression of H2-type genes under stress at any time is upregulated or down-regulated twice as much as the control (Supplementary Figure 2), we selected and collected it for Venn diagram analysis (Figure 2).

### Four Exogenous Hormone Treatments on Branches of the Tea Plant

Tea plants (*Camellia sinensis* var. Sinensis cv. Shuchazao) were collected from the Tea Plant Cultivar and Germplasm Resource Garden in Guohe Town, Anhui Agricultural University. Tea branches having the same growth conditions and divided into four groups, after which they were placed in erlenmeyer flasks containing the same amount of water. After 24 h of adaptation, the water was replaced with 200 mM NaCl solution for Group 1 and with 25% PEG4000 solution for Group 2. Group 3 was treated by smearing 0.25% MeJA on each leaf. Group 4 was treated at 10°C, respectively. The 2nd leaves tissues were collected at seven time points: 0, 6, 12, 24, 36, 42, and 60 h for Group 1; 0, 4, 8, 16, 24, and 36 h for Group 2; at 0, 6, 12, 24, and 36 h for Group 3; 0, 6, 12, 24, 36, 72, and 96 h for Group 4. At each time point, three repeats were performed. Samples were immediately frozen in liquid nitrogen and kept at −80°C for RNA extraction.

### Quantitative Real-Time RT-PCR Analysis

Total RNA was extracted using the FastPure Plant Total RNA Isolation Kit (polysaccharides and polyphenolics-Rich) (Vazyme Cat. RC401-01) according to the manufacturer recommendations. The cDNA were synthesized using PrimeScript RT Reagent Kit Perfect Real Time (TaKaRa, Dalian, China; Code: DRR037A) and stored at −20°C for later use. Verifying PCR product-amplification specificity was determined using the fusion curve (55–95°C). The housekeeping gene was glyceraldehyde-3-phosphate dehydrogenase. The RT-qPCR mixture consisted of 10 μL of CHAMQ SYBR qPCR mixture (Vazyme), 7.4 μL of ddH2O, 0.8 μL of upstream and downstream primers, and 1 μl of cDNA. RT-qPCR reacted in the 96-well optical reaction plates at 95°C for 30 s, followed by 40 cycles at 95°C for 5 s and reaction at 60°C for 30 s PCR product amplification specificity was determined using the melting curve (55–95°C). Three biological replicates and three experimental replicates were applied for each sample. The relative expression values were calculated using the 2^–ΔΔ*Ct*^ method. In each group, untreated branches were used as control. All primers used for RT-qPCR analysis were recorded in Supplementary Table 4.

### Subcellular Location Analysis

The steps of subcellular localization analysis were referred to Yoo et al. (2007), and some modifications were made. *CsMIEL1* was constructed on pUC19-GFP vector and transformed into *Arabidopsis* protoplasts of leaves.

### *In vitro* Ubiquitination Assays

The entire *CsMIEL* and *CsMIEL1-C192S* open reading frame was cloned into the pEGX vector and expressed in *Escherichia coli*, the primers are displayed in Supplementary Table 5. The *in vitro* ubiquitination assays were performed as described elsewhere (Zhao et al., 2012). For the E3 ubiquitin ligase activity assay, recombinant wheat (*Triticum aestivum*) E1 (GI: 136632), human E2 (UBCH5B; 100 ng), and purified *Arabidopsis* ubiquitin with HIS-tag (UBQ14, AT4G02890; 500 ng) were mixed and used for the assay. The mixture was incubated at 30°C for 2 h, boiled at 100°C for 5 min, and frozen at −20°C until the SDS-PAGE analysis. After Western blotting, the reactants isolated by SDS-PAGE were incubated with GST antibody (1:10,000) and observed using ECL chromogenic solution.

### Sequence Alignment and Phylogenetic Analysis

Multiple sequence alignments of the full-length RING proteins were performed by DNAMAN (Version 6.0; Lynnon Corporation, Quebec City, QC, Canada) with default parameters. The phylogenetic tree was constructed with molecular evolutionary genetics analysis (MEGA) software (Version 6.0)^7^ (Tamura et al., 2013), using the neighbor-joining (NJ), minimal evolution (ME), and maximum parsimony (MP) methods and the bootstrap test carried out with 1,000 iterations to test the significance of the nodes.

### Generation of the Overexpressing Transgenic *Arabidopsis thaliana*

*Arabidopsis* ecotype *Columbia (Col-0)* plants were grown in Murashige and Skoog (MS) media at 22°C in long day conditions (16 h of light and 8 h of darkness) and used as wild types and for genetic transformation and other analyses. The *Agrobacterium tumefaciens GV3101* strain was grown in LB media supplemented with 50 μg/mL kanamycin and 20 μg/mL rifampicin. The *CsMIEL1-MYC* and *CsMIEL1-C192S-MYC* construct consisted of the coding sequence under the control of a 35S promoter. Transgenic *Arabidopsis thaliana* organisms were generated using floral-dip transformation (Clough and Bent, 1998). All T0 generation seeds were sown on MS medium containing 15 μg/mL glyphosate for selection. RNA was extracted and reverse transcribed into cDNA for semi-quantitative PCR, and finally four *CsMIEL1-MYC* transgenic lines and *CsMIEL1-C192S-MYC* transgenic lines were obtained. Among them, Line 2, 4, and 6 of CsMIEL1 have high expression level, and Line 8 is low. Line 2, 3, and 4 of *C192S* are high, and Line 1 has low expression level (Supplementary Figure 3). Through the glyphosate selecting of each generation until the T3 generation, PCR verification assays have confirmed that the overexpressing line is homozygous.

### Stress Induction in *Arabidopsis thaliana* Seedlings

Wild-type, *CsMIEL1-MYC*, and *CsMIEL1-C192S-MYC* overexpressing *Arabidopsis thaliana* were sown simultaneously in Petri dishes containing the same volume of MS medium under common growth conditions. After 10 days of pre-culture, the seedling of the same size was selected and treated on the MS medium with 100 mM NaCl (Zhang et al., 2008; Kuki et al., 2020), 200 mM mannitol (Malefo et al., 2020), 100 μm of MeJA (Leon-Reyes et al., 2010), and 10°C (Zhang et al., 2008) for another 10 days. Plants were grown for 10 days under normal conditions after pre-culture as controls (Mock). In all biological replicates, at least 50 plants were used for each treatment, and the error bars represent the standard deviation of replicates.

### Extraction of Anthocyanin Content

Extraction of anthocyanin was extracted according to Jiang et al. (2017) with some modifications. In total, 0.2 g of plant tissue was quick-frozen in liquid nitrogen for preparation for subsequent experiments. During extraction, plant tissues were fully ground with a ball mill and extracted four times through the addition of a 500 μL extraction buffer. The ratio of the buffer was 80% methanol, 1% hydrochloric acid, and 19% water. After each extraction, the homogenate was centrifuged at 12,000 rpm for 10 min and combined. The absorbance values were measured at 530 nm, and each genotype was repeated at least three times.

### Measurement of Plants Growth Index

The plants were stripped from the MS medium, and various growth indicators were measured manually. We recorded the data of all biological replicates for calculating the average and standard deviation. The measurement was all done by one person to control the error.

### Statistical Analysis

For statistical analyses, IBM SPSS Statistics version 19 were use. The Tukey’s and least significant difference (LSD) multiple comparison tests were used to analyze significant differences between pairs. Differences were considered statistically significant when ^∗^*P* < 0.05 and ^∗∗^*P* < 0.01. All the results were based on the average of three parallel experiments.

## Results

### Classification of RING Protein in *Camellia sinensis*

**FIGURE 1:**
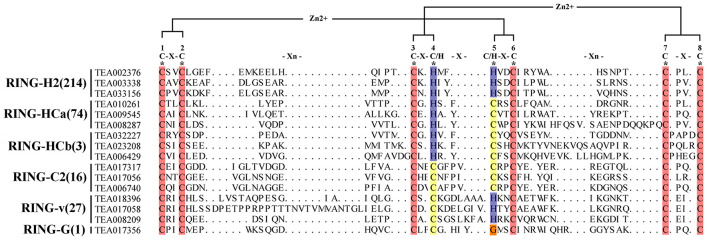
Structural analysis of RING finger domains. Bracketed numbers represent the amount in each RING domain. *Eight conserved docking sites. Red represents amino acid conserved sites, other colors represent different residues.

We downloaded the sequence of the RING family in *Arabidopsis* for analysis of this huge family in the tea plant. After deduplication and comparison, 335 RING-contain proteins were identified. On the basis of the differences in amino acid sequences at these binding sites and the number of amino acids between each site, these 335 domain in tea plant were divided into six categories: RING-H2, RING-HCa, RING-HCb, RING-C2, RING-v, and RING-G. In all types, the amino acid residues at positions 1, 2, 3, 6, 7, and 8 were conserved and consisted of Cys, whereas the amino acid residues 4 and 5 were not conserved. According to the aforementioned classifications, 64% (214) of the RING domain in tea plant were distributed into the RING-H2 group. The metal-ligand positions 4 and 5 are two His residues, which accounted for the name of the RING-H2 group. According to the characteristics of the amino acid residues of metal ligands 4 and 5, the remaining RING types were classified into RING-HCa, HCb, C2, V, and G groups. The differences between HCa and HCb groups corresponded to the number of amino acids between positions 7 and 8. Between metal ligands 7 and 8, the HCa group comprised two and four amino acids, respectively. Few members belonged to the HCb and G groups (only three members and one member, respectively). Similar to *Arabidopsis*, most proteins were the H2 type followed by the HCa type. In contrast to *Arabidopsis thaliana*, among the 477 RING proteins in *Arabidopsis thaliana*, 41 (8.6%) HCb-type proteins were present, whereas only 3 HCbs were present in the tea plants. We downloaded data from the Grape Genome Browser^8^ database and performed the same manual search and identification of the RING domain in grapes to compare the RING-containing protein classifications of different species (Supplementary Table 2). Apple’s data in the table comes from previous reports (Zhang et al., 2008). Among the four species, the distribution of RING domains was consistent; the H2-type domains were the most numerous followed by the HCa-type domains. The number of V-type and C2-type domains in each species was nearly identical, but the number of HCb-type domains differed substantially. Research has speculated that the RING-D domain may only exists in *Arabidopsis thaliana* (Stone et al., 2005). Accordingly, in our search of tea plants and grapes, we did not identify this type of RING domain. But in Apple, a D-type RING domain was found (Zhang et al., 2008). However, no C2-type grape RING domains were found in this selection. Perhaps due to the problem of genome assembly and annotation, the identification in tea plant did not involve S/T-Type, D-type, mH2-type and mHC-type, and the structure of these types was not explained in detail (Figure 1).

**FIGURE 2:**
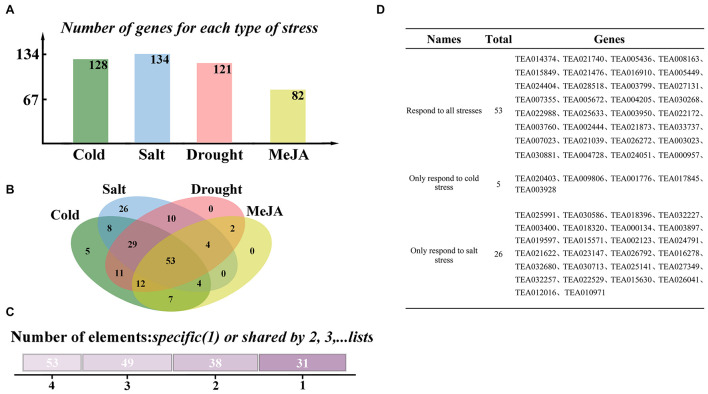
Selection of RING-containing proteins in response to stresses in tea plants. **(A)** Number of genes responding to various types of stresses. **(B)** Venn diagram of genes responding to different stresses. **(C)** The number of genes responding to stresses at the same time. **(D)** List of genes responding to different stresses.

We predict other motifs of RING finger proteins in tea plants. The results indicated that 235 members exhibited no other motifs and contained only the RING finger domain. An additional 17 domains were identified in the remaining RING-containing proteins, such as the WD40 domain, IBR domain, and other motifs with unknown functions (Supplementary Table 3). The WD40 domain is cited as a binding module for pSer/Thr. Additionally, when present in ubiquitin ligase, the WD40 domain may also be related to the phosphorylation of the relevant substrate (Deshaies, 1999; Koepp et al., 1999; Nakayama et al., 2000). The IBR domain is a common Cys-rich region located between two RING domains, but its function is unknown (van der Reijden et al., 1999). The identification of numerous domains suggests that the RING-containing proteins in tea plants may have multiple regulation or regulated mechanisms and perform diverse functions.

### Selection of RING-Containing Proteins Involved in Stress Resistance

To figure out the RING finger genes related to stress responses in tea plants, we analyzed the induced expression difference data of 335 genes provided by TPIA under different stresses. Abiotic stresses included cold, salt, and drought treatments. Responses to biological stress were simulated by detecting the response of plants to exogenous methyl jasmonate (MeJA). Differentially expressed H2-type RING genes were individually compared and analyzed, and the results are presents in Supplementary Figure 2. Figure 2A reports the number of genes responding to various stresses. In total, 128, 134, 121, and 82 genes responded to cold stress, salt stress, drought stress, and exogenous MeJA treatment, respectively. A Venn diagram indicates that 53 genes responded to all stresses (cold stress, drought stress, salt stress, and MeJA treatment), and 31 genes respond to only one form of stress. A total of 26 genes responded only to salt stress, and five responded only to cold stress. However, no gene responded only to drought stress or MeJA treatment (Figures 2B,C). These findings are reported in Figure 2D.

**FIGURE 3:**
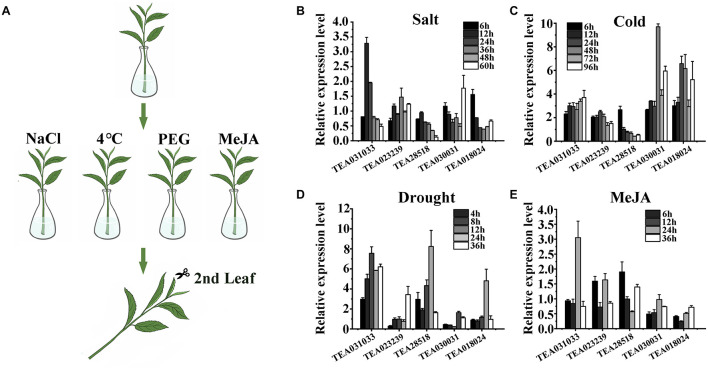
Validation of transcriptomic data by quantitative real-time analysis. **(A)** Schematic of four stress treatments on branches of the tea plant. **(B–E)** Quantitative real-time analysis data of five selected genes subjected to four stresses.

To verify the response of these genes to stress, we treated the branches of tea plants with a low degree of lignification with NaCl, PEG4000, a low-temperature condition, and exogenous MeJA to simulate the four selected stresses. After the treatments were completed, RNA was extracted from the second leaf and reverse transcribed into cDNA for Quantitative real-time RT–PCR analysis (RT-qPCR) (Figure 3A). In the process of designing and RT-qPCR primers, we found that for many genes, the target fragments could not be cloned, or the amplified products were not specific, and most genes could not design primers with an efficiency of 90–110%. This may be caused by genome assembly and annotation issues. Therefore, we selected five genes that met the requirements of amplification efficiency for RT-qPCR: TEA031033, TEA023239, TEA28518, TEA030031, and TEA018024. The results of the RT-qPCR analysis indicated that gene responses to salt and MeJA were relatively low (Figures 3B,E), but responses to drought and cold were more substantial (Figures 3C,D). Among the selected genes, TEA031033 had the strongest response when treated with NaCl for 12 h: the response was 3.28 times that of the control (Figure 3B). After 24 h of MeJA treatment, the expression level of TEA031033 was approximately three times that of the control (Figure 3E), which also represented the most considerable gene response to exogenous MeJA. TEA030031 and TEA018024 responded significantly to cold stress; their strongest response to cold stress were 9.6 and 6.6 times that of the control, respectively (Figure 3C), but TEA030031 had a weak response to salt, drought, and MeJA treatment. Under drought stress, the strongest response values of TEA28518, TEA031033, and TEA018024 were 8.23, 7.55, and 4.8 times that of the control, respectively. These five genes exhibited different levels of response to biotic and abiotic stresses, but TEA031033 had a relatively high level of response to all four types of stress. Therefore, we conducted an in-depth investigation on this gene to identify its specific function in tea plants.

### CsMIEL1 as an E3 Ubiquitin Ligase

**FIGURE 4:**
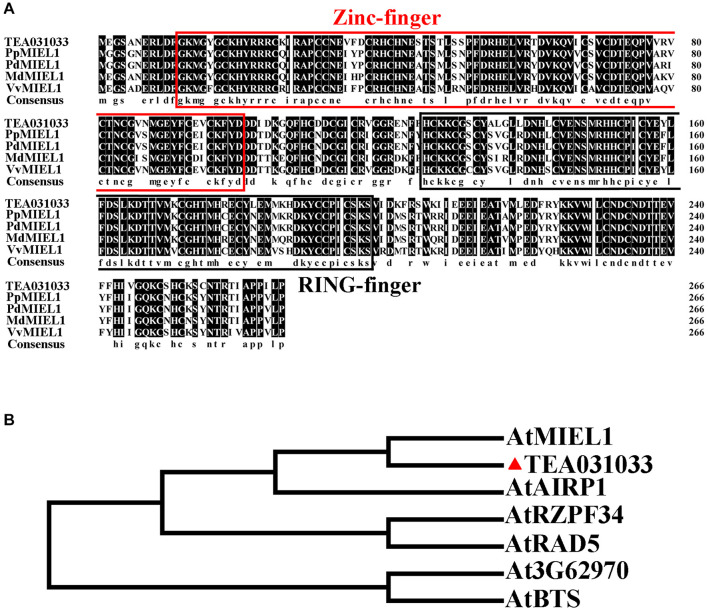
Bioinformatics analysis of CsMIEL1. **(A)** Amino acid sequence alignment of MIEL1 in different species. **(B)** Phylogenetic analysis of TEA031033 and RING-containing proteins in *Arabidopsis*.

We cloned TEA031033 and compared its sequence with that of other species. Our results indicated that TEA031033 has a RING finger domain at the C-terminus and a Zinc-finger domain at the N-terminus (Figure 4A). The phylogenetic analysis results indicated that AtMIEL1 had the highest homology with TEA031033 (Figure 4B). Consequently, TEA031033 was named CsMIEL1. Subcellular localization results show that *CsMIEL1* is localized in the nucleus (Supplementary Figure 4).

**FIGURE 5:**
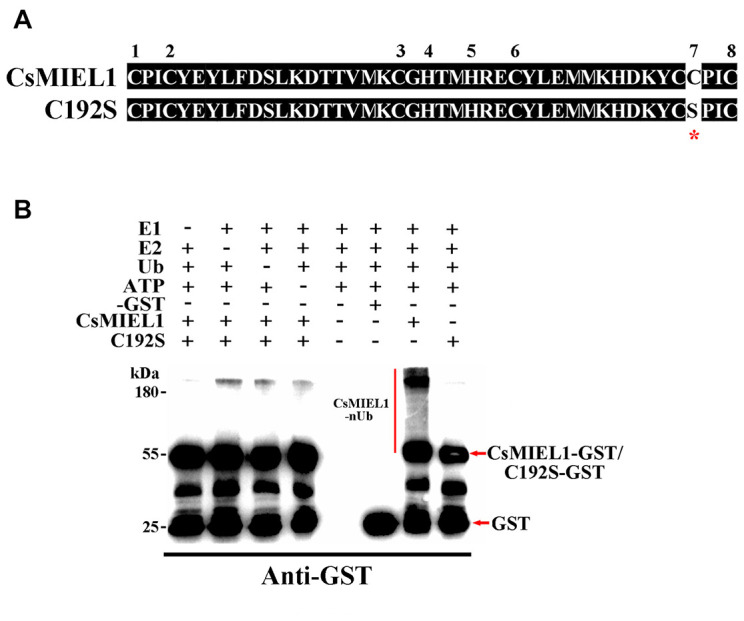
Verification of the activity of CsMIEL1. **(A)** Comparison of the amino acid sequence of CsMIEL1 and CsMIEL1-C192S. **(B)** *In vitro* ubiquitination analysis of CsMIEL1 and CsMIEL1-C192S. *Sited-directed mutagenesis position.

First, to verify the specific function of CsMIEL1 in tea plants, we investigated the potential presence of E3 ubiquitin ligase activity, an *in vitro* ubiquitination assay was conducted. We cloned the open reading frame of the *CsMIEL1* and C192S site-directed mutagenesis, the 192th residue of C192S was changed from Cys to Ser (Figure 5A). Polyubiquitinated CsMIEL1 were found in the presence of ATP, ubiquitin, E1, E2, and CsMIEL1-GST fused proteins using an anti-GST antibody, an absence of any factor had cause CsMIEL1 to not be ubiquitinated. However, even in the presence of all the auxiliary factors, no polyubiquitin CsMIEL1-C192S was detected in assays (Figure 5B). As a result, we concluded that the RING finger family protein CsMIEL1 exhibited E3 ubiquitin ligase activity and that this ubiquitination activity was lost after mutation at position 192. Moreover, we concluded that the complete RING domain is necessary to preserve the E3 ubiquitin ligase activity of the RING finger protein.

### Overexpression of *CsMIEL1* Alters Root Development Under Various Stresses

**FIGURE 6:**
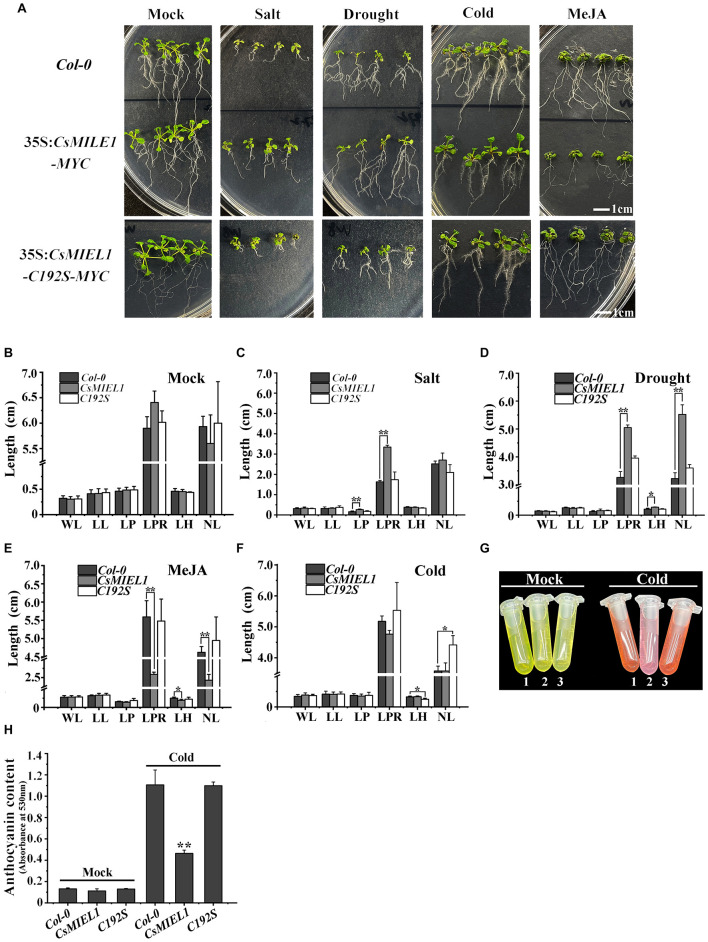
Resistance experiment of transgenic *Arabidopsis* plants to four stresses. **(A)** Four stress treatments for wild-type (*Col-0*), *CsMIEL1*, and *CsMIEL1-C192S* overexpressing *Arabidopsis thaliana* seedlings. **(B–F)** Data on each index of the three types of seedlings under the four stress treatments. Mock, Seedlings without stress treatment; WL, Width of leaf; LL, Length of leaf; LP, Length of petiole; LPR, Length of primary root; LH, Length of hypocotyl; NL, Number of lateral roots. **(G,H)** Anthocyanin concentration of three *Arabidopsis* genotype under cold stress and normal conditions. In all biological replicates, at least 50 plants were used for each treatment, and the error bars represent the standard deviation of replicates. The asterisks indicate the significance level (*P < 0.05, **P < 0.01) based on the LSD’s honestly significant difference test.

To investigate the functions of CsMIEL1, we obtained *35S: CsMIEL1-MYC* and *35S: CsMIEL1-C192S-MYC* overexpressing *Arabidopsis* plants. The four selected stress treatments were performed on the wild-type, *CsMIEL1-MYC*, and *CsMIEL1-C192S-MYC*, respectively (Figure 6A). For the transgenic plant seedlings and wild-type seedlings grown at room temperature for 10 days without any exogenous hormones, no significant differences existed across the growth indicators, including leaf length (LL) and width (WL), hypocotyl length (LH), and root number and length (Figure 6B).

Under salt stress conditions, the growth of both transgenic plant seedlings and wild-type (WT) seedlings was inhibited. The growth of *CsMIEL1-MYC* transgenic plants was less pronounced than that of wild-type and *CsMIEL1-C192S-MYC* plants, which was reflected in the significantly longer length of the petiole (LP) and primary root (LPR) compared with the control (Figure 6C). Similarly, under drought stress, the growth of the transgenic plant and wild-type seedlings was also inhibited, but the length of the primary root (LPR) and hypocotyl (LH) and the number of lateral roots (NL) of *CsMIEL1-MYC* transgenic plants were significantly greater than those of the control and *CsMIEL1-C192S-MYC* (Figure 6D).

The effect of cold treatment on the growth of all seedlings was not particularly notable. The difference in growth indicators between *CsMIEL1-MYC* transgenic plants and WT did not reach a significant level, although its primary root length (LPT) was lower than these of wild-type and *CsMIEL1-C192S-MYC* transgenic plants (Figure 6F). Under low-temperature conditions, the anthocyanin levels of *CsMIEL1-MYC* transgenic plants were significantly lower than these in the control and *CsMIEL1-C192S-MYC* transgenic plants (Figures 6G,H).

The result in Figure 6E showed that *CsMIEL1*-overexpressing plants were hypersensitive to MeJA and that the growth of the root system of the *CsMIEL1-MYC* transgenic plant was significantly inhibited. Additionally, after these three genotypes were treated with exogenous MeJA, the primary root length (LPT), hypocotyl length (LH), and lateral root number (NL) of the *CsMIEL1*-overexpressing plants were shorter than those of the wild-type and *CsMIEL1-C192S-MYC* transgenic plants.

## Discussion

RING finger proteins are vital in plant growth, development, and stress resistance (Deshaies and Joazeiro, 2009). Researchers have thoroughly investigated the RING finger family in *Arabidopsis*; at least 477 RING proteins have been identified (Stone et al., 2005; Yang et al., 2016), and many genes encoding RING domain proteins have been proven to be involved in cold stress (Dong et al., 2006; Suh et al., 2016), heat stress (Lim et al., 2013; Liu et al., 2016), drought stress (Lee et al., 2009; Ryu et al., 2010), salt stress (Qin et al., 2008; Fang et al., 2015), sugar treatment (Huang et al., 2010), and defense responses (Huang et al., 2010; Marino et al., 2019). Although RING proteins have been reported in many species, few studies have explored RING finger family proteins in tea plants. In this study, we could at least find 335 genes encoding RING proteins from tea plants genome. It can be seen that RING finger proteins is a superfamily in the plant kingdom.

In order to understand the role of RING finger proteins in the resistance of tea plants, we used the transcriptome sequencing data provided by the TPIA to select the RING finger genes involved in the resistance of tea plants to biotic and abiotic stresses. By comparing their gene expression differences, a total of 53 H2-type genes encoding RING proteins were found to respond to cold stress, drought stress, salt stress, and MeJA treatments. This also shows that the RING finger proteins family may be widely involved in the anti-stress effects of tea plants. Among them, TEA031033 was selected for function confirmation in this paper, due to its relatively high level of response to all four types of stress.

The protein cloned from TEA031033 is highly conserved with the MIEL proteins reported in other species (Lee and Seo, 2016; An et al., 2017), and named as CsMIEL1. CsMIEL1 contains a Zinc-finger domain at the N-terminus and a RING finger domain at the C-terminus, with 79.78% sequence identity with AtMIEL1 and 83% sequence identity with MdMIEL1. *In vitro* ubiquitination analysis has confirmed that CsMIEL1 has ubiquitin ligase activity, and its 192th amino acid Cys is closely related to its ubiquitination activity. Under exogenous hormones treatments, overexpressing *CsMIEL1* transgenic plants showed a certain degree of resistance to salt stress and drought stress and were hypersensitive to exogenous MeJA. But *C192S* transgenic plants did not show the same response to these stresses as *CsMIEL1*, and most were no different from wild-type.

The lack of understanding of the targets of MIEL1 limits our understanding of the diversity of their responses to stress. E3 ubiquitin ligases often function by interacting with their target proteins. AtMYB30, acts as a positive regulator of the hypersensitive cell death program in plants, and it can interact with AtMIEL1 (Marino et al., 2019). Besides, AtMIEL1 can also change the response to ABA by interacting with AtMYB96 (Lee and Seo, 2016). MdMYB308L is a known target gene identified to interact with MIEL1. It positively regulates cold tolerance and anthocyanin accumulation by interacting with MdbHLH33 and enhancing its binding to MdCBF2 and MdDFR promoters, while apple RING E3 ubiquitin ligase MdMIEL1 decreases the cold tolerance and anthocyanin accumulation promoted by MdMYB308L and directly degrade MdMYB308L (An et al., 2017). The response of *CsMIEL1* overexpressing plants to various stresses indicates the diversity of downstream target proteins. Consequently, the selection of CsMIEL1-interacting proteins is particularly critical for future research. In the previous studies, MIEL1 has a negative regulatory effect on both salt and drought stress, which is contrary to our results, while CsMIEL1’s regulation of cold stress was similar to MIEL1 in apples. Because E3s have a high selectivity for downstream proteins, we speculate that the downstream target protein of CsMIEL1 may not be homologous to the target protein reported in previous studies. We also need a variety of other methods (such as yeast library screening) to screen and identify the target protein.

## Data Availability Statement

The datasets presented in this study can be found in online repositories. The names of the repository/repositories and accession number(s) can be found in the article/Supplementary Material.

## Author Contributions

HR and GM were responsible for using the HMMer search sequence. TL was mainly responsible for the classification and deduplication of sequences. DX was responsible for the remaining work and writing of the manuscript. LG and TX conceived and designed the study. LG drafted the manuscript. All authors contributed to the article and approved the submitted version.

## Conflict of Interest

The authors declare that the research was conducted in the absence of any commercial or financial relationships that could be construed as a potential conflict of interest.

## Publisher’s Note

All claims expressed in this article are solely those of the authors and do not necessarily represent those of their affiliated organizations, or those of the publisher, the editors and the reviewers. Any product that may be evaluated in this article, or claim that may be made by its manufacturer, is not guaranteed or endorsed by the publisher.

## Footnotes

1
https://www.arabidopsis.org

2
http://pfam.xfam.org

3
http://tpia.teaplant.org/

4
http://www.hmmer.org/

5
http://www.ebi.ac.uk/interpro/

6
http://smart.embl.de/

7
http://www.megasoftware.net/

8
https://www.genoscope.cns.fr/externe/GenomeBrowser/Vitis/
