# Supplementary material for: Transcriptome-Wide Analysis and Functional Verification of RING-Type Ubiquitin Ligase Involved in Tea Plant Stress Resistance
Source: Front Plant Sci. 2021 Oct 21;12:733287. doi: 10.3389/fpls.2021.733287 (PMC8568054; doi:10.3389/fpls.2021.733287)
Supplement: Supplementary file 1 [file Data_Sheet_1.docx]

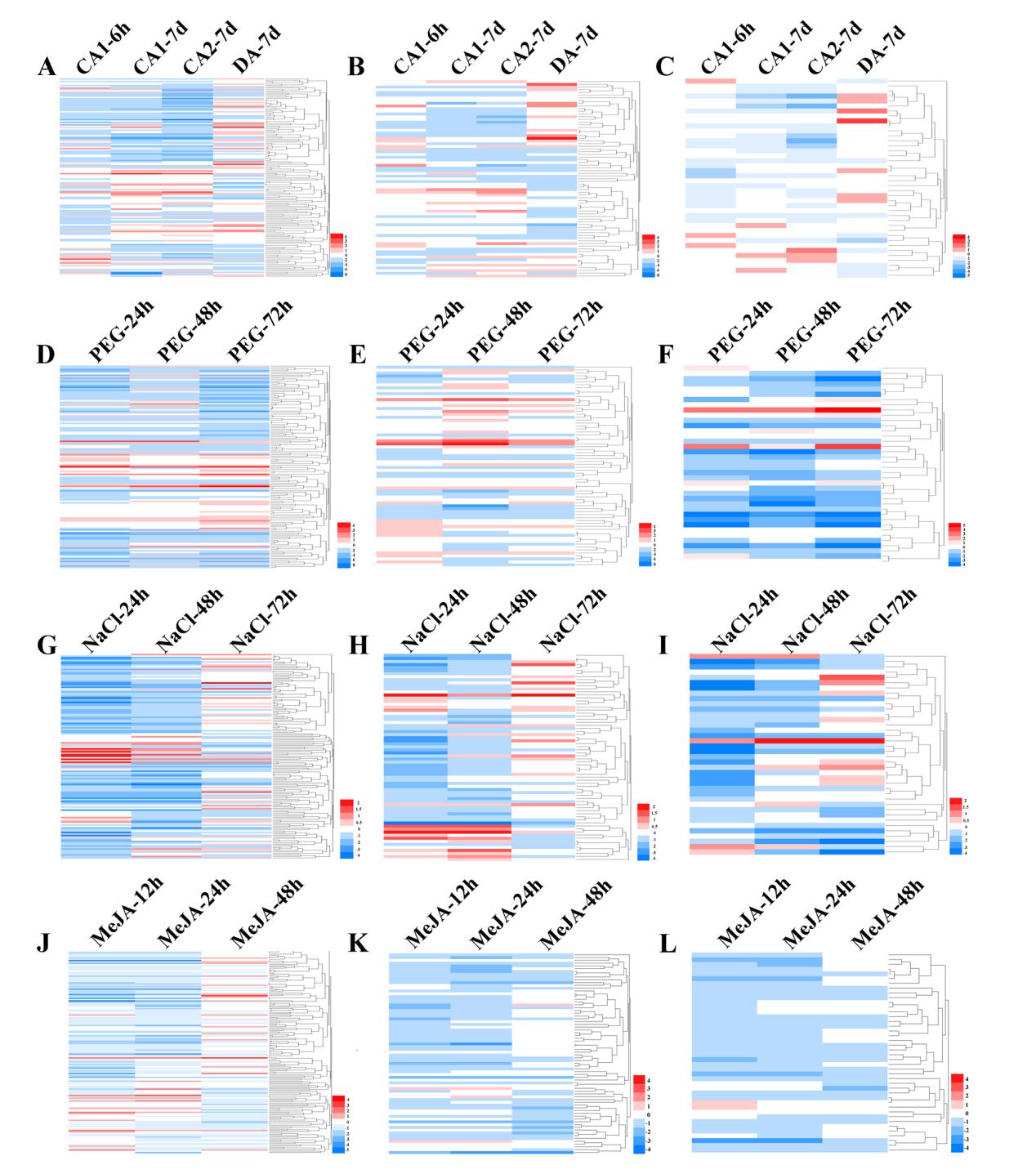


**Supplementary Figure 1. Heat maps of RING finger genes in tea plants in response to four stresses.** A. D. G. J. Response of H2-type RING genes to four stresses. B. E. H. K. Response of HC-type (HCa-type and HCb-type) RING genes to four stresses. C. F. I. L. The response of the remaining ( C2-type, v-type , and G-type) RING genes to the four stresses. The value in the figure is log2 data of treatment versus control.


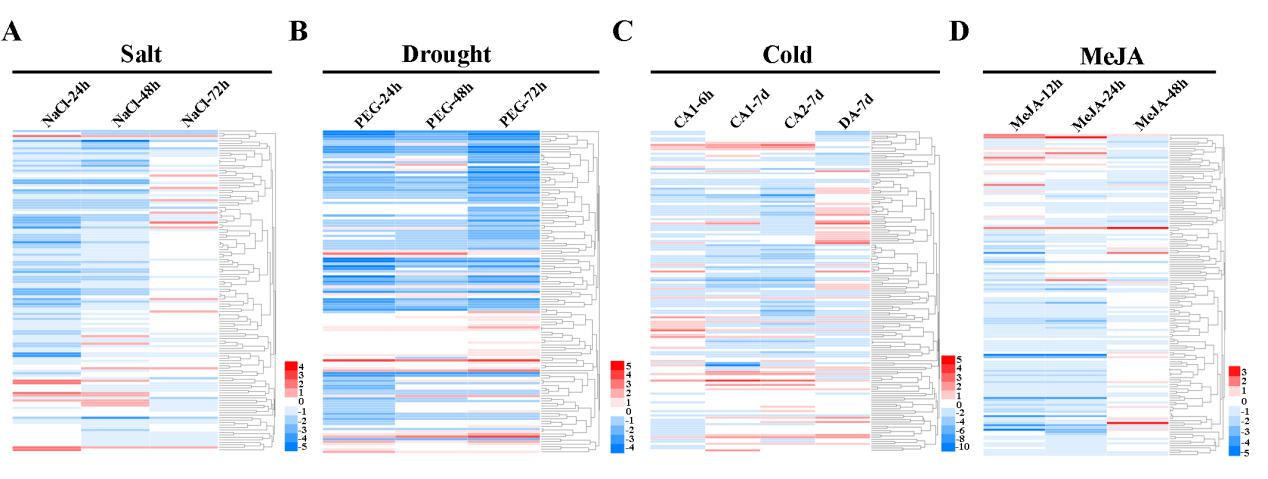


**Supplementary Figure 2. Heat maps of H2-type RING finger genes in tea plants in response to four stresses.** A. Response of H2-type RING genes to salt stress. B. Response of H2-type RING genes to Drought stress. C. The response of H2-type RING genes to the cold stress. D. The response of H2-type RING genes to the biological stress (simulation by exogenous MeJA). The value in the figure is log2 data of treatment versus control.


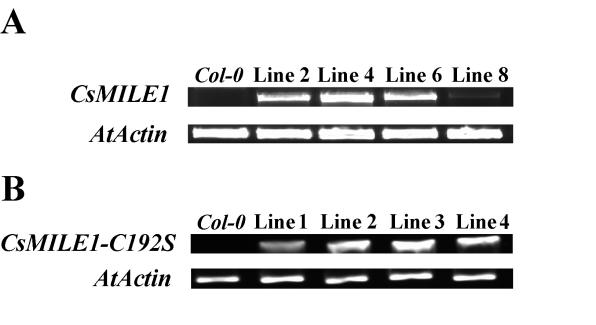


**Supplementary Figure 3. Semi-quantitative PCR analysis of overexpressing *Arabidopsis* plants.** A. Semi-quantitative analysis of four lines of *CsMIEL1* overexpression plants. B. Semi-quantitative analysis of four lines of *CsMIEL1-C192S* overexpression plants.


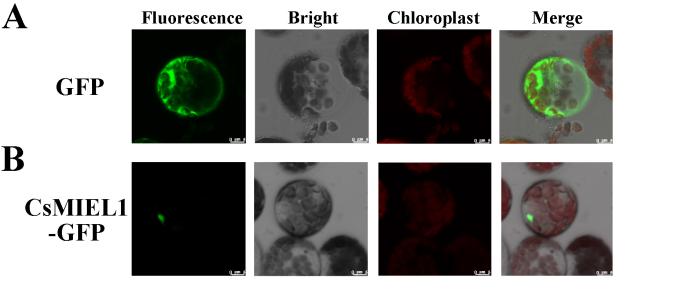


**Supplementary Figure 4.** **Subcellular localization analysis of *CsMIEL1.*** A. Subcellular localization of pUC19 vector. B. Subcellular localization of *CsMIEL1*; Supplementary Figure 4B shows that *CsMIEL1* is located in the nucleus
